# Soy Isoflavones Mitigate High-Fat Diet-Induced Oxidative Stress and Inflammation in the Gut of *Monopterus albus* via Gut Microbiota Remodeling

**DOI:** 10.3390/biology14111586

**Published:** 2025-11-13

**Authors:** Shao Wang, Minglang Cai, Quan Li, Huahong Wei, Yi Hu, Junzhi Zhang

**Affiliations:** Fisheries College, Hunan Agricultural University, Changsha 410128, Chinacml950518@outlook.com (M.C.);

**Keywords:** *Monopterus albus*, high-fat diet, soy isoflavone, gut microbiota, oxidative stress, inflammation

## Abstract

High-fat diets are commonly used in eel aquaculture to reduce protein costs, but excessive fat intake can impair intestine health. This study investigated whether soy isoflavones (SIFs), an emulsifying additive, could mitigate the negative effects of high-fat diets in eel. Six hundred eels (30.00 g) were randomly divided into four groups with three replicates: control (CK, 5.96% fat), high-fat diet (HFD, 11.96% fat), and HFD supplemented with 50 mg/kg (LSF) or 100 mg/kg SIF (HSF) for 8 weeks. Results show that SIF supplementation reversed adverse effects on eels fed high-fat diets, reducing intestine damage (better villi development and lower malondialdehyde levels) and improving antioxidant capacity (higher glutathione, *p* < 0.05). SIFs also increased key antioxidant-related enzyme activities (catalase, superoxide dismutase, *p* < 0.05). Gut microbiota analysis revealed that SIFs restored microbial balance by reducing Proteobacteria (*Pseudomonas*) and increasing Firmicutes (*Lactococcus*), while suppressing harmful metabolic pathways like lipopolysaccharide biosynthesis. These findings demonstrate that dietary SIFs (50 mg/kg) effectively counteract high-fat diet-induced intestine damage and gut dysbiosis in eels, offering a practical nutritional strategy for sustainable aquaculture.

## Simple Summary

High-fat diets are often used in fish farming to reduce costs, but too much fat can harm their growth and health. This study explored whether adding soy isoflavones (SIFs), a natural phytic substance, to high-fat diets could help eels stay healthier. Researchers fed eels different diets: a normal-fat diet, a high-fat diet, and high-fat diet with two levels of SIFs. After eight weeks, eel on the high-fat diet showed signs of intestinal damage. However, dietary supplementation with SIFs boosted their ability to fight oxidative stress and inflammation. SIFs also help balance gut bacteria, reducing harmful microbes while promoting beneficial ones. These changes led to better digestion and overall health. The findings suggest that SIFs could be a valuable additive in eel farming, helping them thrive on high-fat diets while avoiding negative health effects. This could benefit farmers by improving eel production and quality, supporting sustainable aquaculture practices.

## 1. Introduction

Dietary protein presently accounts for the majority of aquaculture costs [1], driving the industry towards alternative energy sources. Concomitantly, uneaten feed and undigested protein excreted in feces contribute significantly to water quality degradation in aquaculture systems [2]. Therefore, developing high-energy feeds has become a major focus in aquatic nutrition research [3], with carbohydrates [4] and lipids [5] serving as primary energy sources. However, the limited ability of most aquatic animals to utilize dietary carbohydrates constrains their application in formulated feeds [6]. Since dietary lipids are generally less expensive than protein, numerous studies have confirmed their protein-sparing effect [7,8], making high-lipid diets a common strategy in aquaculture [3,9]. Studies in fish and crustaceans have shown that appropriate dietary lipid levels can enhance hepatic lipase activity without compromising health [10,11]. Yet, excessive lipid intake can lead to reduced growth performance [12] and an increased feed conversion ratio [10]. Furthermore, increasing feed fat levels significantly increases fat content in *Takifugu rubripes* (Temminck & Schlegel, 1850) [2], with similar results observed in *Scylla serrata* (De Haan, 1983) [13], and *Ctenopharyngodon idellus* (Steindachner, 1866) [14]. In addition, high-fat diets can induce oxidative stress [15], exacerbate tissue damage, and cause intestinal microbiota dysbiosis [9]. Chronic high-fat intake has been shown to increase malondialdehyde (MDA) levels in serum and liver tissues of species such as *Micropterus salmoides* (Lacepede, 1802) [16], *Nibea coibor* (Hamilton, 1822) [17], and *Penaeus vannamei* (Penaeus vannamei Boone, 1931) [18], while also downregulating the expression of antioxidant genes such as *sod* and *gpx*. Given the adverse effects, it is crucial to explore nutritional interventions that can mitigate the negative impacts of high-fat diets.

Soy isoflavones (SIFs), a class of bioactive phytoestrogens predominantly derived from soybeans, have garnered increasing attention in aquatic nutrition due to their multifunctional properties, particularly in enhancing antioxidant capacity and modulating inflammatory responses and lipid metabolism [19,20,21]. In aquaculture, bioactive additives such as SIFs are often necessary in formulated feeds to maintain intestinal health and support metabolic homeostasis, especially under challenging dietary conditions [20,22]. Previous studies have demonstrated that SIF supplementation significantly enhances intestinal antioxidant defense by upregulating the activity of endogenous antioxidant enzymes and lowering levels of oxidative stress markers [23,24]. Moreover, SIFs exhibit notable anti-inflammatory properties, as they can inhibit pro-inflammatory cytokine production and suppress NF-κB activation in intestinal tissues [19,25]. Beyond local effects on the gastrointestinal tract, SIFs also influence gut microbial composition by promoting the growth of beneficial bacteria (*Clostridiales*) and inhibiting potential pathogens, thereby contributing to improved gut barrier function and microbial ecosystem stability [19]. Given their bioactivity and physiological versatility, SIFs represent a promising functional feed additive for mitigating intestinal oxidative stress, inflammation, and microbiota dysbiosis in aquaculture species.

*Monopterus albus* (Zuiew, 1793), commonly known as the rice field eel, is an economically important subtropical freshwater fish widely distributed in East and Southeast Asia [19], with China being its major producer, particularly in the Yangtze River basin where annual production exceeds 300,000 tons according to Chinese Fishery Yearbook. As a carnivorous species that relies predominantly on olfaction for foraging, *M. albus* has been increasingly subjected to high-fat dietary regimens in aquaculture to improve growth and feed efficiency, yet these often exceed their physiological lipid requirements (around 7–8%) and may induce metabolic disorders [9]. Importantly, the gut microbiota plays a central role in regulating host lipid metabolism [26,27]. Research indicates that a high-fat diet induces pathological damage in the intestinal tract of fish and promotes lipid metabolism, thereby increasing the abundance of beneficial microbial communities. These microbial communities effectively mitigate host injury [28,29,30]. Specific microbial communities, particularly those dominated by phyla such as Firmicutes and Bacteroidetes [9], influence lipid homeostasis through multiple mechanisms [31,32]. Additionally, bacterial metabolites such as lipopolysaccharides (LPS) can promote adipogenesis and inflammation [33], further linking microbial ecology to host metabolic health. Given the demonstrated impact of SIFs on oxidative stress and gut microbiota in our previous investigation [19], this study aims to evaluate the protective effects of SIF supplementation on intestinal health in *M. albus* fed high-fat diets, specifically focusing on its modulation of antioxidant capacity, inflammatory responses, and gut microbiota composition, thereby addressing a critical gap in nutritional intervention strategies for sustainable aquaculture.

## 2. Materials and Methods

### 2.1. Experimental Diets

The experimental diets were formulated using the power feeds of eels as the foundation [19]. The main protein sources included fish meal, soybean meal, brewer yeast powder, and poultry by-product meal. Fish oil was used as the principal fat source, while corn starch was utilized as the carbohydrate source and adhesive. Eels were domesticated on a basal diet (CK, 5.96% fat). A negative control diet was prepared as a high-fat feed (HFD, 11.96% fat) according to a previous publication [34]. Two supplementary dietary formulations were developed by supplementing the HFD with two actual concentrations of soy isoflavones: 50 mg/kg SIFs (LSF) and 100 mg/kg SIFs (HSF). The soy isoflavone product (40% purity of soy isoflavone, catalog No. S11165) was provided by Yuanye Biotechnology (Shanghai Yuanye Biotechnology Co., Ltd., Shanghai, China) and mainly consists of soy isoflavones. The compositional ingredients details of the CK and HFD diets are summarized in Table 1. Following initial sieving through a 60-mesh sieve, dietary ingredients were sequentially blended in incremental proportions. The homogenized dry mixture was subsequently combined with fish oil through mechanical mixing. The feed was dried until its moisture content fell below 10% and stored at −20 °C until used. Prior to feeding, the diet was rehydrated by adding 20% water, kneaded into a dough, and then administered. Nutritional composition analysis was performed using standardized analytical protocols: protein content was measured through nitrogen determination via the Kjeldahl method (FP-528 analyzer, Laboratory Equipment Corporation, Livonia, MI, USA), while lipid content was assessed through solvent extraction using the Soxhlet extraction method (SOX406 system, Hanon Future Technology Group Co., Ltd., Jinan, China). Both analytical methods were conducted following Chinese national standards (GB/T 6433-2025 [35] for crude fat and GB/T 6432-2018 [36] for crude protein content determination).

### 2.2. Feeding Management

An experimental trial was carried out at a joint facility in Xihu Town (Changde City, Hunan Province, China), with random assignment of genders. Prior to the experiment, eels underwent a 15-day acclimatization phase. During the initial acclimatization, they were fed earthworms and fish pulp to adapt them to artificial feeding, gradually transitioning until they readily accepted a full fish pulp diet. In the later stage, a mixture of formulated feed and fish pulp (initially at a 1:4 ratio) was provided. The proportion of formulated feed was progressively increased based on their feeding response, until the eels fully adapted to consuming the formulated diet alone. This acclimatization phase was preceded by a 48 h fasting period to ensure metabolic standardization. Subsequently, a total of 600 eels (approximately 30.00 g) were randomly allocated into twelve net cages (2.0 × 2.0 × 1.5 m), with three cages per group, containing fifty individuals per net cage. The feeding regimen consisted of one daily administrations (17:00–18:00) at a rate of 3–4% of their body weight, maintained for an 8-week experimental period. Feed allocation was adjusted weekly according to biomass measurements to maintain appropriate feeding ratios [37]. Throughout the entire experiment, water conditions were consistently maintained at the optimal state for the research subjects: pH levels were kept between 7.1 and 7.5, dissolved oxygen content remained above 6.5 mg/L, the average water temperature was 29.0 °C, and ammonia levels stayed below 0.5 mg/L.

### 2.3. Ethic Statement

All experimental protocols were conducted in compliance with the animal care standards of HUNAU and received ethical approval from the HUNAU Animal Care and Use Committee (grant number 2025EA121).

### 2.4. Sampling

Intestine and intestinal content samples were extracted from three individuals per group for intestine microscope observations, biochemical examination, detection of gene expression levels, and bacterial profile analysis.

### 2.5. Determination of MDA Content and Enzyme Activity

Intestine samples were used to estimate the contents of reduced glutathione (GSH, A006-2-1) and malondialdehyde (MDA, A003-1-1). Also, the activities of catalase (CAT, A007-1-1), superoxide dismutase (SOD, A001-1-2), and total antioxidant capacity (T-AOC, A015-1-2), were measured in the intestine. All of the above indicators were determined using spectrophotometry. Hydrogen peroxide (H_2_O_2_, A064-5-1) content in the intestine was measured using a fluorescence assay method. The assay kits were all acquired from Nanjing Jiancheng Bioengineering Research Institute (Nanjing, China), and the tested procedures were carried out as provided.

### 2.6. Intestine Microscope Observations and Image Fitting

Preparation of the intestine sections followed the procedure described in Supplementary Material S2. The graphical properties of the intestine sections were assessed using ImageJ software (Version 1.53k; National Institutes of Health, Bethesda, MD, USA). Based on the preceding steps, the intestine villi compactness was fitted to measure the extent of intestine villi development. Specifically, rectangular sampling was performed at similar locations of the intestinal sections across all groups, with three random samplings. The gray values of the selected areas (the lamina propria within the mucosal layer), converted by color thresholding, were analyzed.

### 2.7. Gene Expression Detection

Intestine RNA isolation, quality assessment, and cDNA synthesis protocols are detailed in Supplementary Materials S3. Gene-specific primers were designed using Primer 5.0 software (Premier Biosoft, Palo Alto, CA, USA), with *rpl17* serving as the internal control based on established eel sequences from the NCBI. Target genes included oxidative stress-related genes (*nrf2*, *sod*, *gpx1*, *gpx8*, and *cat*), tight junction protein-related genes (*zo1*, *zo2*, *occ*, and *claudin12*), and inflammation-related genes (*nfkb*, *tgfb1*, *tlr5*, *tlr8*, *tnfa*, *il15*, *il8*, and *il1β*). All oligonucleotides, synthesized by Tsingke Biotechnology Co., Ltd. (Shanghai, China), are listed in Table 2 with corresponding sequences and abbreviations. Quantitative PCR was conducted following established protocols [38], with gene expression quantification performed using the 2^−ΔΔCt^ method after confirming primer amplification efficiencies within the 90–110% range.

### 2.8. Amplicon Sequencing and Microbial Community Profiling

Microbiota profiling was conducted through Illumina-based sequencing of the 16S rRNA gene using the paired-end method. The 12 intestinal content samples were processed by Personal Biotechnology Co., Ltd. (Shanghai, China) for high-resolution taxonomic profiling. Sequence data processing (including quality filtering, taxonomic classification, and preprocessing parameters) involves the following detailed steps Supplementary Materials S4: DADA2 was called for quality control, denoising, and chimera removal via the command ‘qiime dada2 denoise-paired’ (Callahan et al., 2017 [39]). Following this, the amplicon sequence variants (ASVs) feature sequences and ASV tables were merged, and the Silva132 database was selected for annotation (Quast et al., 2012 [40]). Additionally, Muscle5 was used for multiple sequence alignment (Edgar, 2022 [41]), followed by sequence trimming using trimal (Capella-Gutiérrez et al., 2009 [42]), the construction of an evolutionary tree by maximum likelihood method using iqtree (Minh et al., 2020 [43]). Furthermore, the relative abundance of ASVs that can be observed in each sample at that sequencing depth is predicted by rarefaction, where a certain number of sequences are randomly selected from each sample separately to achieve a uniform depth.

Microbial community profiling yielded 400164 quality-filtered sequences, averaging 33347 reads per sample. Initial data processing involved filtering low-abundance amplicon sequence variants (ASVs), with taxonomic classification performed using ASVs for enhanced computational analysis. Beta diversity assessment was conducted through Bray–Curtis dissimilarity matrices, followed by dimensionality reduction via Principal Coordinate Analysis (PCoA) and visualization via the core functions in the ggplot2 package (Supplementary Materials S5) [44]. Differential microbial abundance analysis was performed using linear modeling with established thresholds (*p* < 0.05, *fold change* > 1). The network construction utilized four adjacency matrices derived from amplicon sequence variants (ASVs) that met the significance threshold (0.7) of Spearman correlation coefficients across replicate samples. To assess the robustness of the identified modules derived from differential ASV network, a sensitivity analysis was performed by reconstructing the network at a higher similarity threshold (0.8). Moreover, microbial interaction networks were constructed using Random Matrix Theory (RMT) with a similarity threshold of 0.995, revealing non-random co-occurrence patterns, as evidenced by comparative analysis of empirical versus random network properties (Table 3).

### 2.9. Functional Prediction of Microbial Metabolism

The functional potential of the gut microbiota was predicted based on 16S rRNA gene data using Tax4Fun (version 0.3.1). Briefly, the SILVA rRNA database (release 123) was used as a reference to map the obtained ASVs to prokaryotic genomes. The resulting functional profiles were associated with KEGG Orthology (KO) identifiers. Only KEGG pathways categorized under “Metabolism” were extracted and retained for further analysis to focus on metabolic potential. The functional predictions should be interpreted as in silico inferences of metabolic capacity rather than direct measurements.

### 2.10. General Statistical Analyses

All analyses in this study were conducted using the microchat platform (https://mineraltsai.shinyapps.io/shinymicrochat, 7 November 2025). The data in the tables are presented as “mean ± S.E.M (standard error of the mean)”. Parametric data meeting assumptions of variance homogeneity and normal distribution were analyzed through one-way ANOVA with Tukey’s post hoc tests for multiple comparisons. Non-parametric datasets were subjected to a Kruskal–Wallis ran-sum test followed by Dunn’s test for pairwise comparisons. Statistical significance was determined at *p* < 0.05 (Supplementary Materials S6).

## 3. Results

### 3.1. Intestinal Histomorphology Observation and Tight Juction-Related Gene Expression Patterns

**Figure 1:**
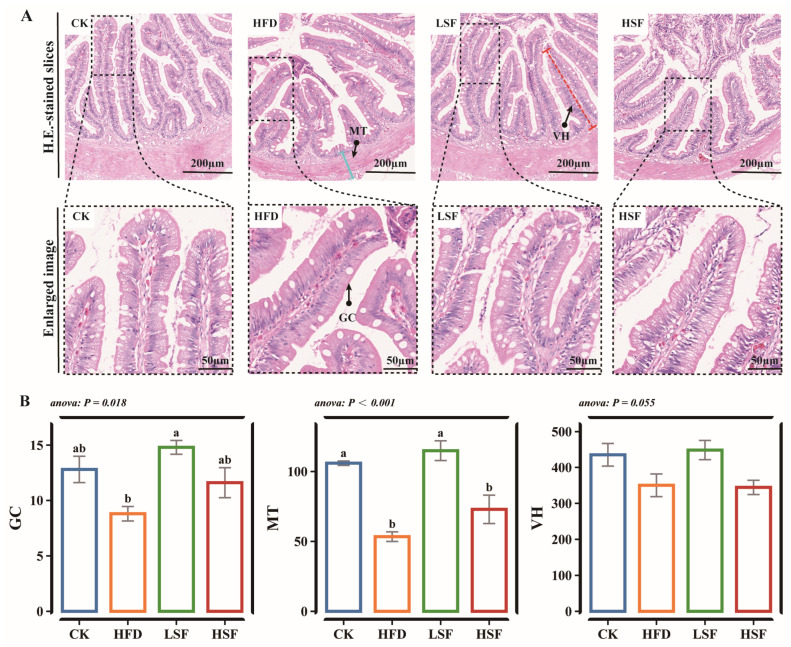
Intestinal histological morphology observation. (**A**) Intestinal H.E.-stained slices (×50). GC, goblet cell (a/root); MT (Blue line), muscle thickness (μm); VH (Red line), villi height (μm). (**B**) Intestinal histological morphology metrics. CK = diet containing 5.96% fat; HFD = diet containing 11.96% fat; LSF = HFD diet supplemented with 50 mg/kg soy isoflavones; HSF = HFD diet supplemented with 100 mg/kg soy isoflavones. Data were analyzed using one-way ANOVA following assumptions of variance homogeneity and normal distribution. Non-parametric datasets were subjected to Kruskal–Wallis ran-sum test followed by Dunn’s test for pairwise comparisons. Values (mean ± S.E.M, *n* = 3 individual fish) in the same row with different superscripts are significantly different (*p* < 0.05).

Based on the histological analysis of intestinal sections, distinct morphological differences were observed among the experimental groups. Compared to the CON group, which exhibited intact mucosal architecture with tall, well-ordered villi and continuous epithelium, the intestines of eels fed HFD diets showed the disorganized epithelial cell arrangement (Figure 1A). Compared to the CON group, the HFD group showed significant intestinal morphology changes, marked by reduced muscle thickness (MT) (*p* < 0.05, Figure 1B). Specifically, the 50 mg/kg SIF intervention effectively reversed these alterations, restoring GC, MT, and villi height (VH) to levels at or above those of the CON group (*p* > 0.05). The HSF group also showed an improvement in MT compared to the HFD group (*p* > 0.05). No notable differences in GC distribution or VH were observed between the HSF and HFD groups.

**Figure 2:**
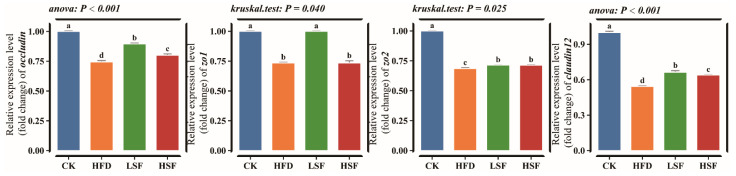
Intestinal tight junction-related gene expression. *zo1*, zonula occludens 1; *zo2*, zonula occludens 2; *occludin*, occludin; *claudin12*, claudin-12. CK = diet containing 5.96% fat; HFD = diet containing 11.96% fat; LSF = HFD diet supplemented with 50 mg/kg soy isoflavones; HSF = HFD diet supplemented with 100 mg/kg soy isoflavones. Data were analyzed using one-way ANOVA following assumptions of variance homogeneity and normal distribution. Non-parametric datasets were subjected to Kruskal–Wallis ran-sum test followed by Dunn’s test for pairwise comparisons. Values (mean ± S.E.M, *n* = 3 individual fish) in the same row with different superscripts are significantly different (*p* < 0.05).

The high-fat diet significantly downregulated the mRNA expression of all tight junction genes (*claudin12*, *occludin*, *zo1*, and *zo2*) compared to the CON group (*p* < 0.01, Figure 2). The LSF group exhibited considerable upregulation of *claudin12*, *occludin*, *zo1*, and *zo2* compared to the HFD group (*p* < 0.05). The HSF group also significantly rescued the expression of *claudin12* and *zo2* (*p* < 0.05).

### 3.2. Intestinal Antioxidant-Related Indicators and Gene Expression Patterns

**Figure 3:**
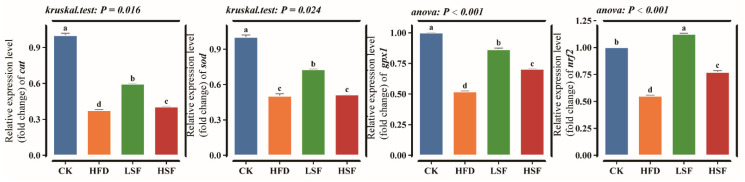
Intestinal antioxidant-related gene expression. *cat*, catalase; *gpx1*, glutathione peroxidase 1; *sod*, superoxide dismutase; *nrf2*, NF-E2-related nuclear factor; CK = diet containing 5.96% fat; HFD = diet containing 11.96% fat; LSF = HFD diet supplemented with 50 mg/kg soy isoflavones; HSF = HFD diet supplemented with 100 mg/kg soy isoflavones. Data were analyzed using one-way ANOVA following assumptions of variance homogeneity and normal distribution. Non-parametric datasets were subjected to Kruskal–Wallis ran-sum test followed by Dunn’s test for pairwise comparisons. Values (mean ± S.E.M, *n* = 3 individual fish) in the same row with different superscripts are significantly different (*p* < 0.05).

High-fat feeding markedly elevated MDA and H_2_O_2_ contents in the intestine while reducing antioxidant capacity, as demonstrated by decreased GSH and T-AOC levels and suppressed SOD and CAT activities (*p* < 0.05, Table 4). High-fat diet supplemented with 50 mg/kg and 100 mg/kg SIF significantly improved all antioxidant and oxidative markers, except for GSH content compared to the HFD group (*p* < 0.05). Consistent with the biochemical findings, the HFD group showed significant suppression in the mRNA expression of *cat*, *gpx1*, *sod*, and the key transcriptional regulator *nrf2* (*p* < 0.05, Figure 3). Supplementation with dietary SIFs at 50 mg/kg considerably upregulated the expression of *cat*, *gpx1*, *sod*, and *nrf2* compared to the HFD group (*p* < 0.05). Eels given 100 mg/kg SIFs exhibited higher expression levels of *cat*, *gpx1*, and *nrf2* (*p* < 0.05).

### 3.3. Intestinal Inflammation-Related Indicators and Gene Expression Patterns

**Figure 4:**
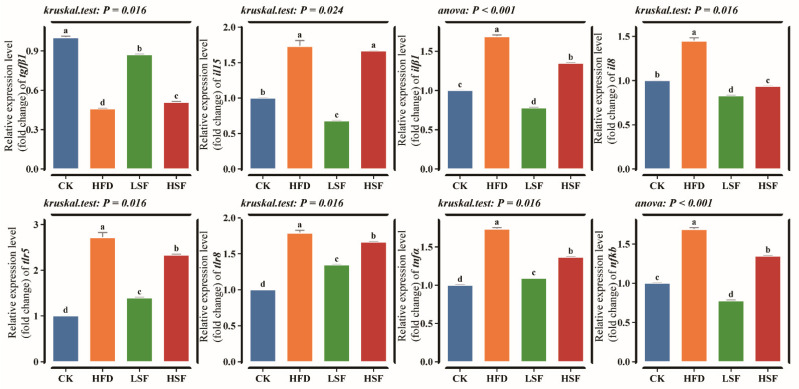
Intestinal inflammation-related gene expression. *nfκb*, nuclear factor kappa B subunit 1; *tgfβ1*, transforming growth factor beta 1; *tlr5*, toll-like receptor 5; *tlr8*, toll-like receptor 8; *tnfα*, tumor necrosis factor alpha; *il15*, interleukin 15; *il8*, interleukin 8; *il1β*, interleukin 1 beta; CK = diet containing 5.96% fat; HFD = diet containing 11.96% fat; LSF = HFD diet supplemented with 50 mg/kg soy isoflavones; HSF = HFD diet supplemented with 100 mg/kg soy isoflavones. Data were analyzed using one-way ANOVA following assumptions of variance homogeneity and normal distribution. Non-parametric datasets were subjected to Kruskal–Wallis ran-sum test followed by Dunn’s test for pairwise comparisons. Values (mean ± S.E.M, *n* = 3 individual fish) in the same row with different superscripts are significantly different (*p* < 0.05).

Eels fed the high-fat diet exhibited a potent pro-inflammatory state, characterized by significant upregulation of *il15*, *il8*, *il1β*, *nfkb*, *tnfα*, *tlr5*, and *tlr8* (*p* < 0.05, Figure 4). Dietary SIF supplementation at 50 mg/kg significantly downregulated *il15*, *il8*, *il1β*, *nfkb*, *tnfα*, *tlr5*, *tgfβ1* and *tlr8* (*p* < 0.05). The HSF diets also significantly ameliorated the expression of most inflammatory markers, including *il1β*, *nfkb*, *tlr5*, *tnfa*, and *tlr8* (*p* < 0.05).

### 3.4. Gut Microbiota Alterations

**Figure 5:**
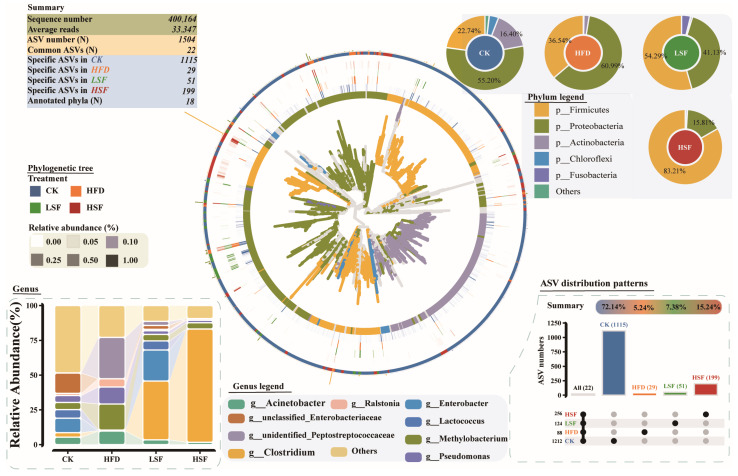
Microbial community structure in the gut of eel. Barcharts of relative abundance at the phylum level explain the distribution of dominant taxa. UpSet plot explains the number of unique and shared microbes in each group. Histogram of relative abundance at the genus level explains the distribution of dominant taxa. Phylogenetic tree was drawn using ggtree. The inner to outer frontal layout represents microbial evolutionary branches (colored by different phyla), microbial rings (colored by different phyla), and relative abundance heatmaps (colored by group). CK = diet containing 5.96% fat; HFD = diet containing 11.96% fat; LSF = HFD diet supplemented with 50 mg/kg soy isoflavones; HSF = HFD diet supplemented with 100 mg/kg soy isoflavones.

**Figure 6:**
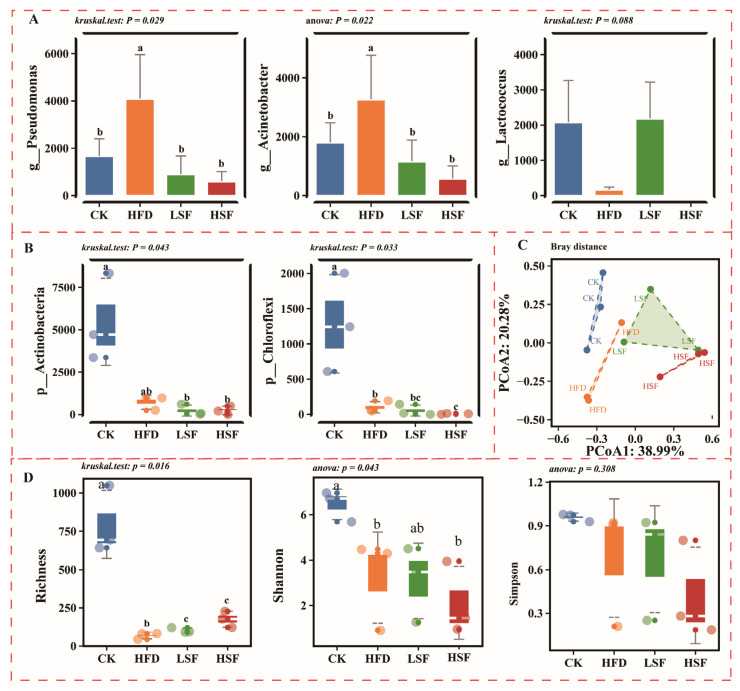
Dominant taxa, alpha and beta diversity of gut microbiota. (**A**,**B**) Significant changes in dominant phyla and genera; (**C**) beta diversity using PCoA analysis based on Bray–Curtis distance. (**D**) Boxplot of Richness index. CK = diet containing 5.96% fat; HFD = diet containing 11.96% fat; LSF = HFD diet supplemented with 50 mg/kg soy isoflavones; HSF = HFD diet supplemented with 100 mg/kg soy isoflavones. Data were analyzed using one-way ANOVA following assumptions of variance homogeneity and normal distribution. Non-parametric datasets were subjected to Kruskal–Wallis ran-sum test followed by Dunn’s test for pairwise comparisons. Values (mean ± S.E.M, *n* = 3 individual fish) in the same row with different superscripts are significantly different (*p* < 0.05).

As seen in Figure 5, a total of 1504 bacterial ASVs were identified across all tested samples. The ASVs counts for each group were 1212 (CK, 72.14%), 88 (HFD, 5.24%), 124 (LSF, 7.38%), and 256 (HSF, 15.24%). Of these, 22 ASVs were common to all treatments, while the numbers of treatment-specific ASVs were 1115 (CK), 29 (HFD), 51 (LSF), and 196 (HSF). Considerable differences could be observed in the Richness and Shannon indexes of gut bacterial profiles between the CK and HFD groups (*p* < 0.05, Figure 6D), of which the Richness and Shannon indexes of the gut microbiota of eels fed the LSF diet were generally higher compared to the HFD group. A 2D diagram effectively captured community variations in the gut microbiota, with significant differences observed between the HFD and SIF-supplemented groups. PCoA1 (38.99%) and PCoA2 (20.28%), based on Bray–Curtis distance, provided a clearer interpretation of these variations (Figure 6C). A phylogenetic tree following sequence trimming and alignment included 18 identified phyla. The gut microbiota was predominantly composed of Proteobacteria, Actinobacteria, Chloroflexi, Fusobacteria, and Firmicutes (Figure 5), of which Chloroflexi were considerably altered in the gut microbial profiles of eels subjected to high-fat feeding (*p* < 0.05, Figure 6A). The gut microbial profiles of eels given a high-fat diet demonstrated decreased Actinobacteria and Chloroflexi abundance compared to the CK group. Conversely, eels fed high SIF-supplemented diets exhibited an increase in Firmicutes and a decrease in Proteobacteria. Taxonomic profiling identified six predominant genera, including *Clostridium* and *Lactococcus* (assigned to Firmicutes), *Methylobacterium*, *Acinetobacter*, *Ralstonia*, *Enterobacter*, and *Pseudomonas* (categorized as Proteobacteria). Of note, *Pseudomonas* and *Acinetobacter* abundance significantly increased in the gut microbiota of the HFD group (Figure 6B), while decreasing in the SIF-supplemented treatments. In addition, high-fat feeding led to a decreased *Lactococcus* in the gut microbial profiles of eels than that of the CK treatment, which was the opposite in the 50 mg/kg SIF-supplemented treatments.

### 3.5. Differential Analysis and Specific-Phenotypes Biomarkers Mining

**Figure 7:**
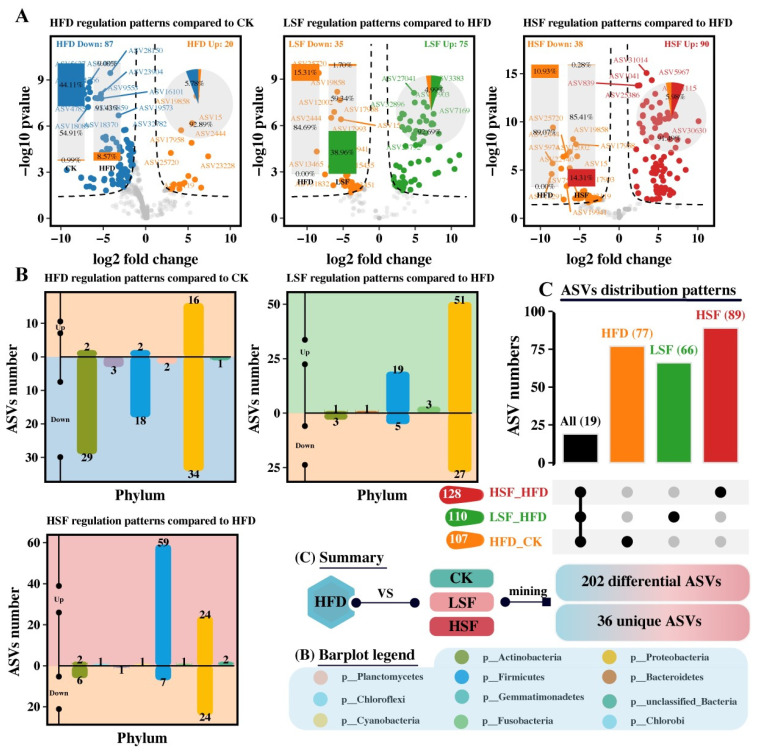
Microbial community differential analysis in the gut of eel. (**A**) The differential ASVs in the HFD and SIF-supplemented groups compared to the CK group; (**B**) distribution of differential ASVs at the phyla level; (**C**) distribution of differential ASVs at the species level. CK = diet containing 5.96% fat; HFD = diet containing 11.96% fat; LSF = HFD diet supplemented with 50 mg/kg soy isoflavones; HSF = HFD diet supplemented with 100 mg/kg soy isoflavones.

Further profiling of gut microbial profiles revealed that the gut of eels given the HFD diet exhibited 87 downregulated ASVs and 20 upregulated ASVs compared to the CK treatment. In contrast, eels on SIF-supplemented diets presented 75 (LSF) and 90 (HSF) upregulated ASVs, along with 35 (LSF) and 38 (HSF) downregulated ASVs (Figure 7A). The upregulated ASVs presented in the HFD group were mainly assigned to Proteobacteria, whereas downregulated ASVs were mostly classified into Actinobacteria, Firmicutes, and Proteobacteria as opposed to the pairwise comparison of the HFD and SIF-supplemented treatments (Figure 7B). Of note, a total of 202 differential ASVs were identified between the CK, and SIF-supplemented treatments compared to the HFD treatment (Figure 7C), in which 36 ASVs were substantially affected by dietary high fat or SIF supplementation.

**Figure 8:**
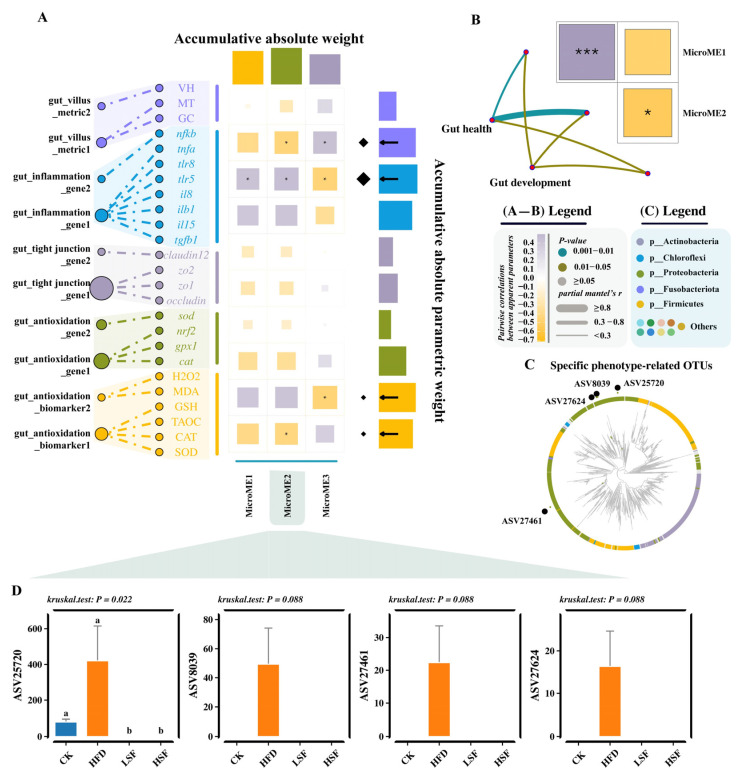
Potential regulatory patterns in eel. (**A**) Mantel’s test results of the interactions between the module eigengenes extracted from the abundance matrix of 88 differential ASVs and the module eigenvectors extracted from the two phenotypes matrix. (**B**) Mantel’s test results of the interactions between the two phenotypes matrix and the module eigengenes extracted from the abundance matrix of 88 differential ASVs. (**C**) Correlation analysis results based on Spearman’s coefficient for the interactions between the module eigengenes extracted from the abundance matrix of 88 differential ASVs and the secondary module eigenvectors extracted from the two phenotypes matrix. (**D**) Boxplot of identified biomarkers. CK = diet containing 5.96% fat; HFD = diet containing 11.96% fat; LSF = HFD diet supplemented with 50 mg/kg soy isoflavones; HSF = HFD diet supplemented with 100 mg/kg soy isoflavones. Data were analyzed using one-way ANOVA following assumptions of variance homogeneity and normal distribution. Non-parametric datasets were subjected to Kruskal–Wallis ran-sum test followed by Dunn’s test for pairwise comparisons. Values (mean ± S.E.M, *n* = 3 individual fish) in the same row with different superscripts are significantly different (*p* < 0.05). * denotes significance at the level of *p* < 0.05; *** denotes significance at the level of *p* < 0.001.

Three modular eigenvectors (MEs) were identified and taxonomically assigned to the abundance matrix derived from the 36 differential ASVs screened (Figure 7C). Meanwhile, the phenotypic data-derived matrix was extracted into 10 eigengenes, all of which were clustered into two types (“Gut health” and “Gut development”). Based on the proceeding step, Mantel’s test, used for dissimilarity matrices, revealed strong correlations (*r* > 0.3, *p* < 0.05) between the “Gut health” module and microbial modular eigen 2 (ME2), through phenotypic matrix eigenvector decomposition and combination (Figure 8A,B). Specifically, the “gut_antioxidation_biomarker1” module (SOD, T-AOC, CAT, and GSH) in “Gut health” and “gut_villus1” module (MT and GC) in “Gut development” displayed stronger negative correlations with ME2 derived from the differential ASV abundance matrix (Figure 8C). Meanwhile, the “gut_inflammation_gene2” module (*nfkb*) showed the opposite patterns. Notably, ME2 and its strong association with host phenotypes were consistently recovered in a sensitivity analysis using a more stringent network construction threshold (0.8), underscoring the robustness of this finding (Supplementary S1). Across our network constructions, the ME2 module was consistently associated with four microbial taxa, which were identified as robust contributors, classified as *Sphingomonas* (ASV25720), *Methylobacterium* (ASV8039 and ASV27624), and *Serratia* (ASV27461) within the Proteobacteria phylum (Figure 8C,D). Of note, the abundance of all four differential ASVs derived from Proteobacteria increased in the gut of eels fed HFD diets and decreased in eels fed diets supplemented with SIFs both at 50 mg/kg and 100 mg/kg.

### 3.6. Molecular Ecological Network Analyses

**Figure 9:**
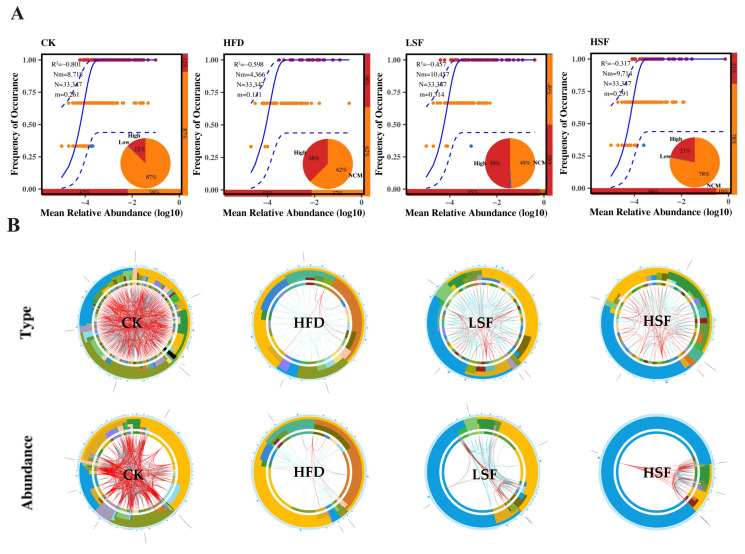
Molecular ecological network analysis in the gut microbiota of eel. (**A**) Community assembly process evaluation based on a neutral community model. A higher R^2^ represents a better fit of the neutral model, which means a greater contribution of stochastic processes to the construction of the community. (**B**) Circos plot for visualizing microbial interactions. The first row is plotted based on microbial species, and the second row is plotted based on abundance. The bands are, from outside to inside, phylum, class, order, family, genus, and species. Red edges represent positively correlated microbial interactions. Green edges represent positively correlated microbial interactions. CK = diet containing 5.96% fat; HFD = diet containing 11.96% fat; LSF = HFD diet supplemented with 50 mg/kg soy isoflavones; HSF = HFD diet supplemented with 100 mg/kg soy isoflavones.

**Figure 10:**
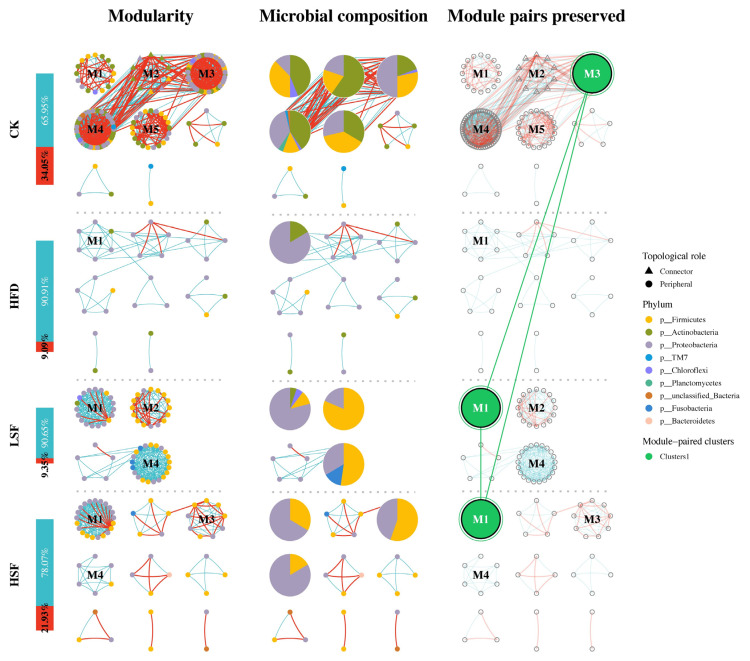
Modularity and ecological roles of molecular ecological network analysis. Specific modules of microbial networks were extracted, highlighting the abundance composition at the phyla level. Modules represent assemblages of microbes that can perform specific ecological functions. The modules of microbial network from each group were analyzed for similarity by Fisher’s test, and they were considered similar if the differences were found to be significant. Similar categories of modules were grouped into module clusters and colored accordingly. This allowed retention of microbial network modules and the potential ecological functions that could be performed under different treatments to be explored. CK = diet containing 5.96% fat; HFD = diet containing 11.96% fat; LSF = HFD diet supplemented with 50 mg/kg soy isoflavones; HSF = HFD diet supplemented with 100 mg/kg soy isoflavones.

The neutral community model revealed poor fits for the gut bacterial profiles across all treatments, suggesting that the community assembly was primarily driven by deterministic processes (Figure 9). The predominant phyla kept in all networks remained Proteobacteria, Actinobacteria, Chloroflexi, Firmicutes, and Fusobacteriota (Figure 9 and Figure 10). High modularity was observed in all networks, which were divided into multiple sub-modules with eight (CK), eight (HFD), four (LSF), and nine (HSF) sub-modules, respectively (Table 4). The gut microbial profiles of eels given the CK diet exhibited the most complex network, as demonstrated by the highest average degree (*avgK*), average clustering coefficient (*avgCC*), average path distance (*GD*), and number of nodes and edges. Moreover, 50 modular pairs were identified, and a total of three individual modules and three modular pairs with considerable variations were observed to be retained across groups, accounting for 6.00% and grouped into one module cluster (Figure 9 and Figure 10). High-fat feeding contributed to the absence of the functional modules assigned to the microbial network of the CK group, while SIF-supplemented diets simply kept a few modules allocated to identified modular-paired clusters. All networks predominantly supported co-existence over co-exclusion, with positive correlations constituting 65.95–90.91% of the potential inter-species interactions analyzed (Table 4). The number of negative-linked interactions varied, with the lowest proportion of negative correlations (9.09%) observed in the network of the HFD group, while the SIF-supplemented increased in negative interactions.

### 3.7. Functional Annotation Prediction

**Figure 11:**
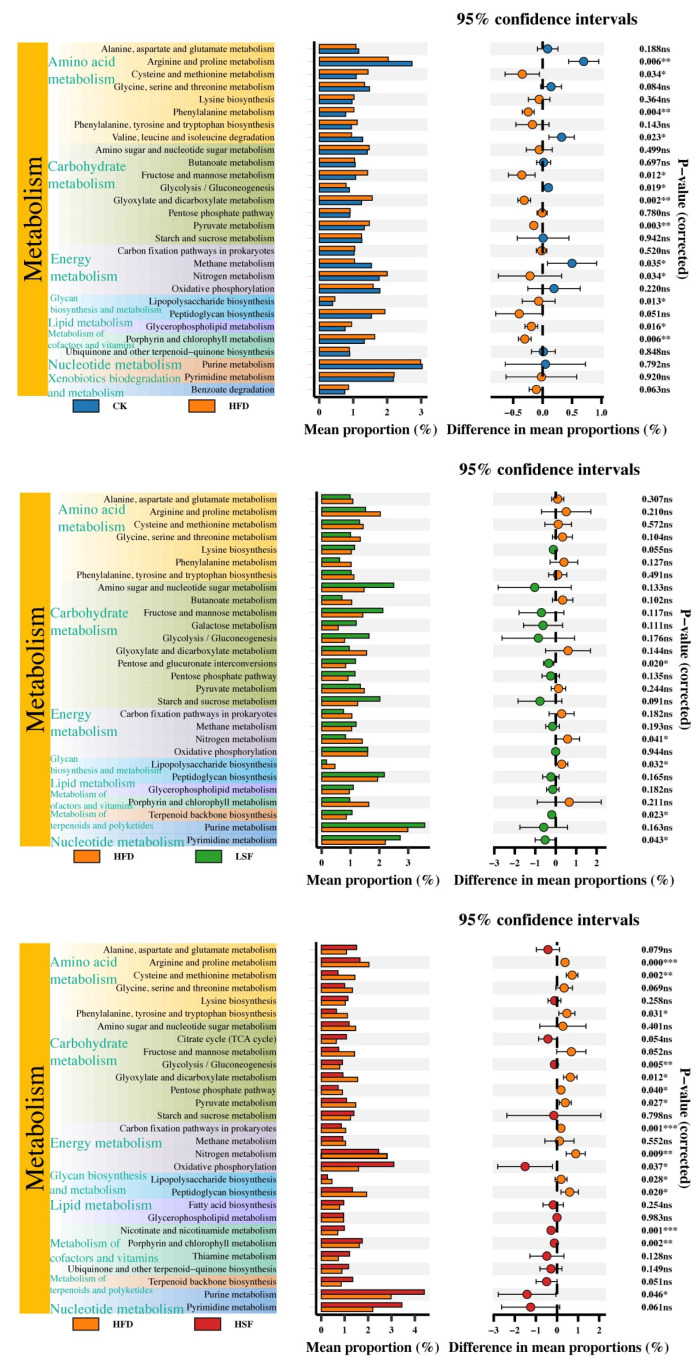
Microbial functional prediction in the gut of eels. * denotes significance at *p* < 0.05; ** denotes significance at *p* < 0.01; *** denotes significance at *p* < 0.001; ns indicates no significance (*p* > 0.05).

Functional annotation of metabolic pathways revealed 264 KO3 categories derived from 9 KO2-level metabolic pathways, with minimum functional abundance reaching 0.8% (Figure 11). The high-fat diet significantly activated cysteine and methionine metabolism, phenylalanine metabolism, fructose and mannose metabolism, glyoxylate and dicarboxylate metabolism, pyruvate metabolism, glycerophospholipid metabolism, nitrogen metabolism, porphyrin and chlorophyll metabolism, and lipopolysaccharide biosynthesis (*p* < 0.05) while significantly inhibiting arginine and proline metabolism, valine, leucine, and isoleucine degradation, glycolysis/gluconeogenesis, and methane metabolism (*p* < 0.05). Moreover, dietary SIFs at 50 mg/kg in a high-fat diet contributed to activating pentose and glucuronate interconversions, terpenoid backbone biosynthesis, and pyrimidine metabolism (*p* < 0.05), while Tax4Fun functional prediction indicates reduced abundance of LPS synthesis-related functions in the SIF supplementation group (*p* < 0.05). Additionally, dietary SIFs at 100 mg/kg activated glycolysis/gluconeogenesis, oxidative phosphorylation, nicotinate and nicotinamide metabolism, porphyrin and chlorophyll metabolism, and purine metabolism (*p* < 0.05), while inhibiting arginine and proline metabolism, cysteine and methionine metabolism, phenylalanine, tyrosine, and tryptophan biosynthesis, glyoxylate and dicarboxylate metabolism, pentose phosphate pathway, pyruvate metabolism, carbon fixation pathways in prokaryotes, peptidoglycan biosynthesis, nitrogen metabolism, and lipopolysaccharide biosynthesis (*p* < 0.05).

## 4. Discussion

Dietary lipids play crucial physiological roles in aquatic species, contributing to cellular membrane integrity, energy supply, and essential fatty acid provision that support growth and development [45]. However, excessive dietary fat intake often leads to metabolic disorders, including fat accumulation, oxidative stress, and inflammatory responses, ultimately impairing intestinal and hepatic health. This aligns with previous studies in aquatic animals, such as rice field eel [9], grass carp [14], and largemouth bass [16], where high-fat diets were shown to cause lipid deposition and disrupt intestinal barrier function [46,47]. In the present study, it was observed that high-fat feeding induced significant intestinal histomorphological alterations in eels, characterized by disrupted epithelial architecture, reduced villus height, decreased goblet cell count, and thinning of the muscularis layer. These structural impairments are consistent with findings in grass carp and largemouth bass, which exhibited similar enteropathy changes under high-lipid dietary stress [48]. Furthermore, these morphological alterations were accompanied by the marked downregulation of key tight junction genes (*claudin12*, *occludin*, *zo1*, and *zo2*), indicating compromised gut barrier integrity reported in high-fat-fed largemouth bass, where intestinal permeability increased, alongside reduced expression of tight junction proteins [47]. Of note, supplementation with soybean isoflavone, particularly at 50 mg/kg, effectively ameliorated these alterations, restoring intestinal morphology and enhancing the expression of tight junction proteins. This is in agreement with studies in mammals and aquatic species, where dietary flavonoids were shown to attenuate high-fat diet-induced gut damage [49,50]. Conversely, some studies have reported that not all phytochemicals exert protective effects at every dosage. In our previous investigation, high doses of soybean isoflavones at 2500 mg/kg failed to improve gut morphology and even induced slight epithelial irritation [19], underscoring the importance of appropriate dosing. Our results demonstrate that SIFs, particularly at a moderate dose of 50 mg/kg, effectively counteract high-fat-induced intestinal impairment, likely through their anti-inflammatory and antioxidant properties, as evidenced in other models [51,52,53].

Consistent with intestinal structural changes, the high-fat diet induced a state of pronounced oxidative stress, as evidenced by elevated MDA and H_2_O_2_ levels, and suppressed activities of key antioxidant enzymes (SOD and CAT). Such findings have been found in other aquatic animals, e.g., largemouth bass [47], tilapia [50], and common carp [3], where high-lipid diets similarly increased lipid peroxidation and impaired antioxidant function. Correspondingly, mRNA expression of *cat*, *gpx1*, *sod*, and the transcription factor *nrf2* was significantly downregulated in the HFD group, indicating suppression of the endogenous antioxidant system, which has also been observed in high-fat-fed juvenile largemouth bass [16] and hybrid grouper [54]. Dietary SIF supplementation markedly attenuated oxidative damage, enhancing antioxidant enzyme activities and upregulating the expression of antioxidant-related genes. This restorative effect mirrors results from studies using flavonoid-like substances in aquatic animals. Dietary flavonoid-like supplements have been shown to upregulate Nrf2 mRNA and its downstream antioxidant genes, consistent with activation of the Nrf2 pathway [55,56]. Conversely, some studies have noted that not all flavonoid-like supplements yield dose-dependent benefits, as excessive polyphenol intake may paradoxically provoke pro-oxidant effects under certain conditions [19,21,23,51]. Our results clearly demonstrate that SIFs, particularly at 50 mg/kg, enhance intestinal antioxidant defense primarily through activation of the Nrf2-Keap1 pathway. In parallel, high-fat feeding triggered a robust pro-inflammatory response, with upregulation of inflammatory cytokines *il1β*, il8, il15, and *tnfα*, and signaling molecules *nfkb*, *tlr5*, and *tlr8*. Similar inflammatory activation has been reported in the intestine of largemouth bass [47], grass carp [48], and mice [49] fed high-fat diets, where TLR/NF-κB signaling was identified as a key mediator. SIF supplementation at 50 mg/kg substantially suppressed the expression of these inflammatory markers, indicating potent anti-inflammatory effects. This is consistent with studies in mammals and fish, where isoflavones inhibited NF-κB nuclear translocation and reduced pro-inflammatory cytokine production [23,25,57]. Given the close interplay among intestinal barrier function, oxidative stress, inflammation, and gut microbiota homeostasis, we further hypothesize that the protective effects of SIFs may also involve microbial regulation. In the following section, we explore how SIF modulates the gut microbial community and its potential role in ameliorating high-fat-induced metabolic and immunological disturbances in eels.

Gut health represents a critical interface between intestinal microbiota and host physiological status alterations [58,59,60]. In this study, our findings demonstrate that SIF supplementation modulated gut microbial composition in high-fat-fed eel. Microbial diversity, encompassing both taxonomic richness and abundance [61], was significantly influenced by dietary interventions. Previous studies have established that high-lipid diets induce microbial dysbiosis [9,62], affecting antioxidant capacity, immune function, and tissue morphology in aquatic species [17,63]. The observed health improvements following SIF supplementation may be mediated through microbial community restructuring [44]. However, previous studies have only outlined shifts in the abundance profiles of animals given a high-fat diet, without providing clear explanations, and the findings remain limited and lacking in logical clarity. Principal coordinate analysis revealed distinct clustering patterns, with a clear separation between HFD and SIF-supplemented groups. The increased richness index in SIF-treated groups suggest enhanced microbial diversity, indicating partial restoration of gut microbiota homeostasis. Taxonomical annotation identified Proteobacteria, Actinobacteria, Chloroflexi, Firmicutes, and Fusobacteriota as dominant phyla, with *Clostridium and Lactococcus* (Firmicutes)*, Methylobacterium, Acinetobacter, Ralstonia, Enterobacter*, *and Pseudomonas* (Proteobacteria) emerging as key genera potentially involved in these microbial shifts. The experimental results align with previous studies in aquatic species, demonstrating that elevated dietary lipid levels can compromise host health through the perturbation of crucial microbial taxa [9,62]. Notably, as the level of dietary SIF supplementation increased, the abundances of *Acinetobacter* and *Pseudomonas* were significantly reduced, while the relative abundance of *Lactococcus* increased. *Acinetobacter* and *Pseudomonas* are typically considered conditionally pathogenic and are often associated with aquatic diseases in aquatic species [64,65,66]. *Lactococcus* could metabolize polysaccharides, with the monosaccharides and other metabolites produced potentially benefiting the host [67,68], which may explain the enhanced growth performance of eel fed SIF diets.

Key microbes have been shown to play significant parts in elucidating the potential causes of variations in functional taxa [69]. It was observed that eel given SIF-supplemented diets presented numerous differential ASVs, further highlighting the specific variations between the SIF-supplemented treatments and the HFD treatment. Notably, a total of 36 differential ASVs were common across all eels given high-fat diets when compared to the CK group, primarily within the phyla Proteobacteria and Firmicutes, as outlined in the primary taxa of the gut microbial profiles. Lipopolysaccharide production is frequently associated with Proteobacteria [70], which can compromise the intestinal mucosal barrier and increase intestinal permeability [71]. This indirectly suggests that SIF supplementation may promote eel health via modulating the gut microbial profiles. Therefore, further investigation into the factors influencing bacterial diversity in response to variations in high-fat and SIF-supplemented diets for eel is needed. One could find that ME2 was primarily influenced by specific amplicon sequence variants, which identified *Sphingomonas* (ASV25720), *Methylobacterium* (ASV8039), and *Serratia* (ASV27461) as the main contributors, whose regulating patterns were the same as those of the corresponding genera. It is worth noting that identification of the ME2 module and its key taxa was not an artifact of a single analytical parameter, as it was consistently identified in sensitivity analyses, thereby increasing our confidence in these results. Current evidence indicates that intestinal microbial imbalance, particularly affecting nutrient metabolic processes, significantly influences host health outcomes [9,69]. The gut microbial profiles play a pivotal role in host nutrient metabolism through their extensive genetic repertoire for metabolic processes [44,72]. Dietary interventions, including SIF supplementation, significantly influenced microbial metabolic functions and community structure. Our findings reveal that high-fat diets specifically enhanced nitrogen metabolism and lipopolysaccharide biosynthesis pathways. The reduced abundance of ASV27461 in SIF-supplemented groups correlated with decreased lipopolysaccharide production, potentially mitigating gut inflammation and oxidative stress associated with Gram-negative bacterial components [73,74]. Collectively, our results suggest that the ME2 module and its constituent taxa may represent a stable microbial feature associated with intestinal health. The reproducibility of this module under varying analytical parameters strengthens the hypothesis that these taxa could play an important role in mediating the protective effects of SIFs.

Microbial community assembly can be interpreted through neutral-based theoretical frameworks, though analysis revealed limited applicability to eel intestinal microbiota, indicating a predominance of deterministic processes [75]. Within the relatively isolated gut environment, microbial taxa exhibit distinct ecological trajectories through conditional or homogeneous selection mechanisms [76], emphasizing the crucial role of interspecies relationships over abiotic factors in community dynamics. Microbial community stability is maintained through complex interaction networks rather than individual behaviors [77], with ecological networks serving as fundamental structures for these interspecies relationships [78]. Microbial communities form complex ecological networks through interdependent relationships, as most species cannot survive independently [79]. Our results reveal decreased network complexity in high fat-supplemented groups, characterized by weakened node-edge connectivity. Network modularity, reflecting the degree of sub-module compartmentalization [80], demonstrated the structural importance of dominant microbial populations. The gut microbial profiles of eel in the SIF-supplemented groups exhibited unique network configurations, with three primary module clusters and eight novel modules, suggesting ecological niche diversification and enhanced community stability. Microbial network stability is maintained through species interactions, with negative correlations generally promoting ecosystem resilience [77,79]. The observed increase in negative interactions among microbial species in SIF-supplemented groups indicates enhanced community stability and resistance to environmental perturbations.

## 5. Limitations and Future Perspectives

Although this study confirmed that soy isoflavone supplementation enhances antioxidant capacity and gut health in eels, limitations remain. The inference regarding Nrf2 pathway activation is primarily based on the upregulation of relevant mRNA transcripts (*nrf2*, *sod*, and *cat*). To definitively confirm pathway activation, future studies should incorporate protein-level analysis: quantitative detection of Nrf2, Keap1, and antioxidant enzymes via Western blotting, combined with immunohistochemical assessment of Nrf2 nuclear translocation.

## 6. Conclusions

In conclusion, our results demonstrate that dietary soybean isoflavone supplementation alleviates high-fat diet-induced intestinal damage by enhancing antioxidant capacity, suppressing inflammatory responses, and restoring gut barrier integrity in *Monopterus albus*. These beneficial effects are likely mediated through the modulation of gut microbiota composition and ecological networks, particularly by promoting the abundance of beneficial taxa and suppressing potential pathogens. These findings provide new insights into the nutritional intervention of high-fat diet-induced metabolic disorders in aquatic species.

## Figures and Tables

**Table 1:** Feed formulation (dried mass).

Ingredients (%)	CK	HFD
Fish meal	40.00	40.00
Soy protein concentrate	16.00	16.00
Poultry by-product meal	3.00	3.00
Brewer yeast powder	5.00	5.00
Fish oil	2.20	8.20
Microcrystalline cellulose	10.26	4.26
Corn starch	20.00	20.00
Choline	0.50	0.50
Premix ^a^	1.00	1.00
Ca(H_2_PO_4_)_2_	2.00	2.00
Antioxidant	0.01	0.01
Anti-mildew agent	0.03	0.03
Total	100.00	100.00
Proximate nutritional analysis (%)		
Crude protein	41.36	41.26
Crude lipid	5.96	11.96

^a^ Premix: The premix used in this study is consistent with that used in previous research [19].

**Table 2:** Information of primers used in this study.

Gene	Forward (5′-3′)	Reverse (5′-3′)	Amplification Efficiency (%)	Source
*rpl17*	GTTGTAGCGACGGAAAGGGAC	GACTAAATCATGCAAGTCGAGGG	96.34	XM_020587712.1
*cat*	GTCCAAGTCTAAGGCATCTCC	CTCCTCTTCGTTCAGCACC	102.11	XM_020624985.1
*gpx1*	TGTGAATGGGAAGGATGCC	CCTGCTGTAACGCTTGAACG	103.51	XM_020607739.1
*sod*	AGCTGGCTAAGTTCTCATTCAC	GCAGTAACATTGCCCAAGTCT	99.67	XM_020598413.1
*nrf2*	CTTCAGACAGCGGTGACAGG	GCCTCATTCAGTTGGTGCTT	91.14	XM_020596409.1
*zo1*	GGCATCATCCCCAACAAA	GCGAAGACCACGGAACCT	108.42	XM_020621576.1
*zo2*	AGCCGAGGTCGCACTTTA	GCTTTGCTTCTGTGGTTGAT	106.88	XM_020615114.1
*occ* *ludin*	TGTCGGGGAGTGGGTAAA	TCCAGGCAAATAAAGAGGCT	105.03	XM_020599328.1
*claudin12*	TCACCTTCAATCGCAACG	ATGTCTGGCTCAGGCTTATCT	92.55	XM_020607277.1
*nfkb*	ACCCTACCGTGACACTAACCT	TGCCGTCTATCTTGTGGAAT	96.32	XM_020616319.1
*tgf* *β* *1*	AACCCACTACCTCACTACCCG	GCCGAAGTTGGAAACCCT	104.17	XM_020623392.1
*tlr5*	TGTAGCCAACTGTGCCTTCCG	CACATTCATGCCGAGCACCAG	98.76	XM_020617011.1
*tlr8*	GTGATGAGAAATCTGCGAGTG	GAGGTTATCTACCAGCGGGAC	107.29	XM_020596483.1
*tnfa*	TTTCAAGGAGGGCTGGTTCT	CTTGACCAGCGCATCACTGT	93.88	XM_020622780.1
*il15*	AGAAATGCCCCATCTCCA	CCCTGTCTCCGTCTTGTTG	101.36	XM_020622724.1
*il8*	TACTGGTTCTGCTTACTGTCGC	CAAATCTTTTGCCCATCCCT	95.47	XM_020597077.1
*il* *1β*	GAGATGTGGAGCCCAAACTT	CTGCCTCTGACCTTCTGGACTT	104.05	KM113037.1

Note: *rpl17*: ribosomal protein L17; *cat*, catalase; *gpx1*: glutathione peroxidase 1; *sod*, superoxide dismutase; *nrf2*: NF-E2-related nuclear factor; *zo1*: zonula occludens 1; *zo2*: zonula occludens 2; *occludin*: occludin; *claudin12*: claudin-12; *nfκb*: nuclear factor kappa B subunit 1; *tgfβ1*: transforming growth factor beta 1; *tlr5*: toll-like receptor 5; *tlr8*: toll-like receptor 8; *tnfα*: tumor necrosis factor alpha; *il15*: interleukin 15; *il8*: interleukin 8; *il1β*: interleukin 1 beta.

**Table 3:** Topological roles in microbial network analyses.

Items	Group			
	CK	HFD	LSF	HSF
Empirical network				
Similarity threshold (*St*)	0.995	0.995	0.995	0.995
Node number (*N*)	155	31	61	56
Edge number (*N*)	1028	55	310	187
Positive edges (%)	65.95	90.91	90.65	78.07
Negative edges (%)	34.05	9.09	9.35	21.93
Average degree (*avgK*)	13.265	3.548	10.164	6.679
Module number (*N*)	8	8	4	9
Average clustering coefficient (*avgCC*)	0.763	0.576	0.763	0.789
Modularity (*M*)	0.559	0.613	0.638	0.55
Average path distance (*GD*)	6.509	4.027	1.896	1.726
Random network				
Average clustering coefficient (*avgCC*)	0.136 ± 0.006	0.131 ± 0.006	0.225 ± 0.006	0.226 ± 0.006
Modularity (*M*)	0.151 ± 0.004	0.359 ± 0.005	0.158 ± 0.005	0.190 ± 0.005
Average path distance (*GD*)	2.309 ± 0.042	2.766 ± 0.016	2.077 ± 0.003	2.344 ± 0.007

Note: CK = diet containing 5.96% fat; HFD = diet containing 11.96% fat; LSF = HFD diet supplemented with 50 mg/kg soy isoflavones; HSF = HFD diet supplemented with 100 mg/kg soy isoflavones.

**Table 4:** Intestinal biochemical indicators.

Items	Group				ANOVA
	CK	HFD	LSF	HSF	*p*
MDA (nmol g^−1^ protein)	2.12 ± 0.02 ^c^	5.21 ± 0.01 ^a^	1.06 ± 0.00 ^d^	3.94 ± 0.04 ^b^	*p* < 0.001
H_2_O_2_ (mmol g^−1^ protein)	9.92 ± 0.04 ^c^	19.02 ± 0.17 ^a^	6.06 ± 0.01 ^d^	14.64 ± 0.05 ^b^	*p* < 0.001
GSH (µmol g^−1^ protein)	7.50 ± 0.09 ^a^	2.87 ± 0.10 ^c^	6.11 ± 0.15 ^b^	3.23 ± 0.19 ^c^	*p* < 0.001
SOD (U mg^−1^ protein)	1.02 ± 0.01 ^a^	0.34 ± 0.02 ^d^	0.78 ± 0.00 ^b^	0.75 ± 0.00 ^c^	0.016
CAT (U mg^−1^ protein)	1.36 ± 0.02 ^b^	0.61 ± 0.03 ^d^	1.54 ± 0.00 ^a^	1.02 ± 0.00 ^c^	*p* < 0.001
T-AOC (mmol g^−1^ protein)	0.05 ± 0.00 ^b^	0.01 ± 0.00 ^d^	0.06 ± 0.00 ^a^	0.04 ± 0.00 ^c^	0.016

Note: MDA, malondialdehyde; H_2_O_2_, hydrogen peroxide; GSH, glutathione; CAT, catalase; SOD, superoxide dismutase; T-AOC, total antioxidant capacity; CK = diet containing 5.96% fat; HFD = diet containing 11.96% fat; LSF = HFD diet supplemented with 50 mg/kg soy isoflavones; HSF = HFD diet supplemented with 100 mg/kg soy isoflavones. Data were analyzed using one-way ANOVA following assumptions of variance homogeneity and normal distribution. Non-parametric datasets were subjected to Kruskal–Wallis ran-sum test followed by Dunn’s test for pairwise comparisons. Values (mean ± S.E.M, *n* = 3 individual fish) in the same row with different superscripts are significantly different (*p* < 0.05).

## Data Availability

The original data presented in the study are openly available at https://github.com/mineraltsai/manuscript_FM/blob/a133234ef4236b53914394524cdc1887b20348c5/data%20for%20biology.zip, accessed on 20 September 2025.

## Supplementary Materials

The following supporting information can be downloaded at https://www.mdpi.com/article/10.3390/biology14111586/s1.
