# Supplementary material for: Soy Isoflavones Mitigate High-Fat Diet-Induced Oxidative Stress and Inflammation in the Gut of Monopterus albus via Gut Microbiota Remodeling
Source: Biology (Basel). 2025 Nov 13;14(11):1586. doi: 10.3390/biology14111586 (PMC12650275; doi:10.3390/biology14111586)
Supplement: Supplementary file 1 [file biology-14-01586-s001.zip › biology-3896515-supplementary.pdf]

Supplementary Figure 1

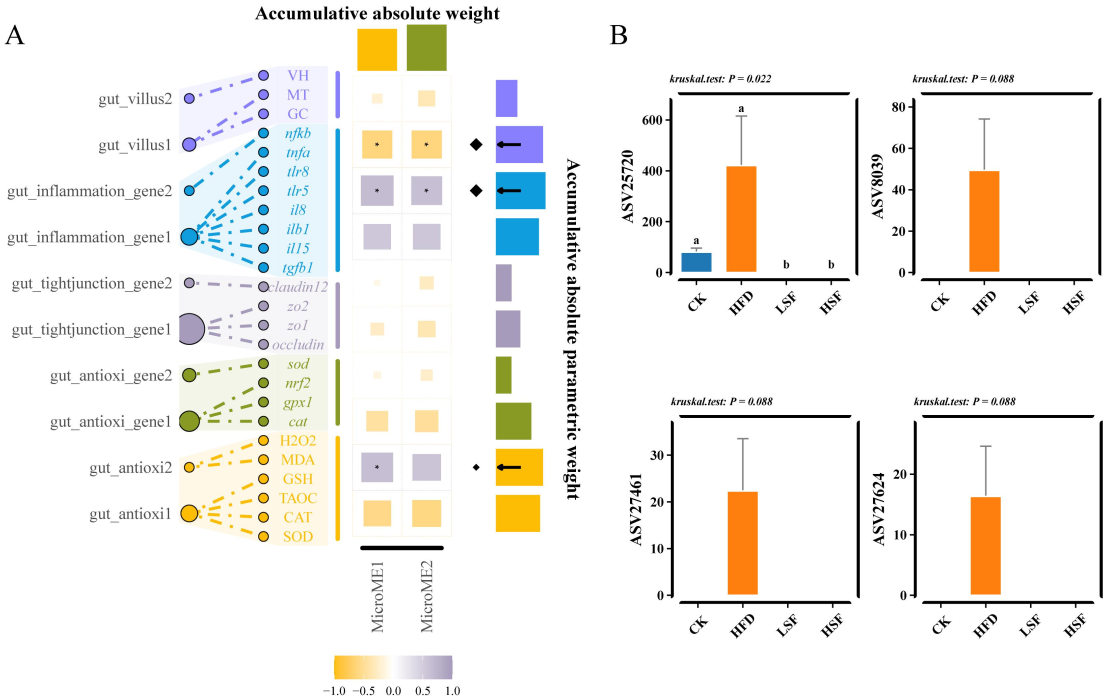

Supplementary material 2

Fresh intestine of eel were fixed in formalin solution for 30-50 minutes. the fixed tissue was dehydrated in 50%, 70%, 80%, 95%, and 100% alcohol for 30 minutes. after that, they were placed in a 1:1 mixture of anhydrous ethanol and xylene for 2 hours, xylene for 1.5 hours, xylene for 1.5 hours. After the clearing process. The fixed tissue was baked in a 1:1 mixture of paraffin and xylene at 40 ℃ for 40 minutes, followed by translucent waxing using paraffin I for 30 minutes and paraffin II for 40 minutes. The oven temperature was raised to 60 ℃, and the pure wax was changed three times, each time for 1-2 hours. The 10-μm paraffin sections were deparaffinized with xylene I for 20 minutes and xylene II for 10 minutes, and then put into various levels of alcoholic solution for 2 minutes and distilled water for 2 minutes, and then immersed in hematoxylin staining solution for 40 minutes, and then rinsed several times in distilled water until they became bluish, and then examined by microscopy. The paraffin sections that passed the microscopic examination were immersed in various levels of alcohol for 2 minutes, then in eosin solution for 20 to 30 seconds, then in 95%, pure alcohol I and pure alcohol II for 3 minutes, and finally sealed with neutral gum for microscopic observation of the tissue structure.

Paraffin section with 10-μm thickness were stained using hematoxylin and eosin (H.E.), as well as microscopically photographed.

Supplementary material 3

The total RNA from the hepatopancreas was isolated using Monzol Reagent Kit (Monad, Wuhan, China). The quantity and quality of the extracted RNA were assessed by agarose gel electrophoresis at 1% and spectrophotometric analysis at 260 and 280 nm. Subsequently, the total RNA was reversely transcribed into cDNA using MonScript™ RTIII All-in-One Mix with dsDNase (Monad, Wuhan, China). The following procedures was carried out: (1) 37 ℃, 2 min; (2) 55 ℃, 15 min; (3) 85 ℃, 5 min. The reaction products were stored at -80 °C.

Supplementary material 4

DADA2 was called for quality control, denoising, and chimera removal via the command `qiime dada2 denoise-paired` (Callahan et al., 2017). Following this, the amplicon sequence variants (ASVs) feature sequences and ASV tables were merged, and the Silva132 database was selected for annotation (Quast et al., 2012). Additionally, Muscle5 was used for multiple sequence alignment (Edgar, 2022), followed by sequence trimming using trimal (Capella-Gutiérrez et al., 2009), the construction of an evolutionary tree by maximum likelihood method using iqtree (Minh et al., 2020). Furthermore, the relative abundance of ASVs that can be observed in each sample at that sequencing depth is predicted by rarefaction, where a certain number of sequences are randomly selected from each sample separately to achieve a uniform depth.

References

Callahan BJ, Mcmurdie PJ, Holmes SP. Exact sequence variants should replace operational taxonomic units in marker-gene data analysis. The ISME Journal 2017;11:2639-43.

Capella-Gutiérrez S, Silla-Martínez JM, Gabaldón T. Trimal: A tool for automated alignment trimming in large-scale phylogenetic analyses. Bioinformatics 2009;25:1972-3.

Edgar RC. Muscle5: High-accuracy alignment ensembles enable unbiased assessments of sequence homology and phylogeny. Nature Communications 2022;13:6968.

Minh BQ, Schmidt HA, Chernomor O, Schrempf D, Woodhams MD, Von Haeseler A, Lanfear R. Iq-tree 2: New models and efficient methods for phylogenetic inference in the genomic era. Molecular Biology and Evolution 2020;37:1530-4.

Quast C, Pruesse E, Yilmaz P, Gerken J, Schweer T, Yarza P, Peplies J, Glöckner FO. The silva ribosomal rna gene database project: Improved data processing and web-based tools. Nucleic Acids Research 2012;41:D590-D6.

Supplementary material 5

Comparison	ANOSIM		Adonis		MRPP	
	r	P	F	P	∂	P
Whole data	0.716	0.001	9.743	0.001	0.66	0.001
CK vs HFD	0.693	0.004	10.033	0.004	0.4	0.003
CK vs LSF	0.702	0.004	7.825	0.004	0.419	0.004
CK vs HSF	0.963	0.004	21.794	0.004	0.31	0.003
HF vs LSF	0.47	0.004	5.231	0.004	0.48	0.004
HF vs HSF	0.817	0.004	12.908	0.004	0.371	0.004

Note: Three different permutation tests were performed (MRPP, ANOSIM and Adonis) on the basis of Bray–curtis distance. Multiple response permutation procedure (MRPP). Analysis of similarity (ANOSIM). Permutational multivariate analysis of variance (Adonis or Permanova).

Supplementary material 6

Figure 1B-Intestinal Morphology	**Test statistic (F or χ^2)**	**df**	**p-value**	**Statistical method**
GC	F = 6.16993464052287	3, 8	0.0177669456151398	one-way ANOVA
MT	F = 19.7072198810684	3, 8	0.000472308109737985	one-way ANOVA
VH	F = 3.88448787705008	3, 8	0.0554170875130362	one-way ANOVA
Figure 2-Expression of tight junction-associated genes in the gut				
occludin	F = 169.763069844312	3, 8	1.39901752861611e-07	one-way ANOVA
zo1	χ^2 = 8.33333333333333	3	0.0396023552075643	Kruskal–Wallis rank-sum test
zo2	χ^2 = 9.35897435897436	3	0.0248799450280187	Kruskal–Wallis rank-sum test
claudin12	F = 494.213973154607	3, 8	2.0371961691332e-09	one-way ANOVA
Table3-Intestinal antioxidant-related indicators				
SOD	χ^2 = 10.3846153846154	3	0.0155643974585932	Kruskal–Wallis rank-sum test
CAT	F =	3, 8	1.8450926789	one-way

	506.677656319339		7096e-09	ANOVA
T-AOC	χ^2 = 10.3846153846154	3	0.0155643974585932	Kruskal–Wallis rank-sum test
GSH	F = 261.464819979265	3, 8	2.54644140658275e-08	one-way ANOVA
MDA	F = 6292.2997171157	3, 8	7.92370410661659e-14	one-way ANOVA
H2O2	F = 4034.09014633884	3, 8	4.68522419355444e-13	one-way ANOVA
Figure 3-Expression of antioxidant-related genes in the gut				
cat	χ^2 = 10.3846153846154	3	0.0155643974585932	Kruskal–Wallis rank-sum test
sod	χ^2 = 9.46153846153846	3	0.0237440664767199	Kruskal–Wallis rank-sum test
gpx1	F = 966.658168856267	3, 8	1.40805528711238e-10	one-way ANOVA
nrf2	F = 674.74486718998	3, 8	5.90038316100834e-10	one-way ANOVA
Figure 4-Gene expression associated with intestinal inflammation				
tgfβ1	χ^2 = 10.3846153846154	3	0.0155643974585932	Kruskal－Wallis rank-sum test
il15	χ^2 = 9.46153846153846	3	0.0237440664767199	Kruskal–Wallis rank-sum test
ilβ1	F = 1011.42621238015	3, 8	1.17545844335304e-10	one-way ANOVA
il8	χ^2 = 10.3846153846154	3	0.0155643974585932	Kruskal–Wallis rank-sum test
tlr5	χ^2 = 10.3846153846154	3	0.0155643974585932	Kruskal－Wallis rank-sum test
tlr8	χ^2 = 10.3846153846154	3	0.0155643974585932	Kruskal－Wallis rank-sum test
tnfα	χ^2 = 10.3846153846154	3	0.0155643974585932	Kruskal–Wallis rank-sum test
nfkb	F = 1011.42621238015	3, 8	1.17545844335304e-10	one-way ANOVA
Figure 6A&B-Significant changes in dominant phyla and genera				
g__*Pseudomonas*	χ^2 = 9.56410256410257	3	0.028659105	Kruskal–Wallis rank-sum test

g__Lactococcus	χ^2 = 6.54951690821257	3	0.087730002	Kruskal–Wallis rank-sum test
g__Acinetobacter	F = 5.238859	3, 8	0.022124131	one-way ANOVA
p__Actinobacteria	χ^2 = 8.12820512820513	3	0.0434350841537281	Kruskal–Wallis rank-sum test
p__Chloroflexi	χ^2 = 8.74358974358975	3	0.0329015914281892	Kruskal–Wallis rank-sum test
Figure 6D-Richness index				
Richness	χ^2 = 10.3846153846154	3	0.0155643974585932	Kruskal–Wallis rank-sum test
Shannon	F = 4.34022049698083	3, 8	0.0430148631332078	one-way ANOVA
Simpson	F = 1.4135523343613	3, 8	0.308352427130334	one-way ANOVA
Figure 8D-identified biomarker index				
ASV25720	χ^2 = 9.59760956175299	3	0.0223153131349173	Kruskal–Wallis rank-sum test
ASV8039	χ^2 = 6.54545454545455	3	0.0878870230026216	Kruskal–Wallis rank-sum test
ASV27461	χ^2 = 6.54545454545455	3	0.0878870230026216	Kruskal–Wallis rank-sum test
ASV27624	χ^2 = 6.54545454545455	3	0.0878870230026216	Kruskal–Wallis rank-sum test
